# Why and When ICD Leads Are Extracted: Does the ICD Lead Model Influence Lead Survival?

**DOI:** 10.3390/medicina61111899

**Published:** 2025-10-23

**Authors:** Andrzej Kutarski, Wojciech Jacheć, Paweł Stefańczyk, Łukasz Tułecki, Tomasz Kukulski, Dorota Nowosielecka

**Affiliations:** 1 2nd Department of Cardiology, Faculty of Medical Sciences in Zabrze, Medical University of Silesia, 40-055 Katowice, Poland; a_kutarski@yahoo.com (A.K.); wjachec@sum.edu.pl (W.J.); tkukulski@sum.edu.pl (T.K.); 2 Department of Cardiology, The Pope John Paul II Province Hospital of Zamość, 22-400 Zamość, Poland; 3 Department of Cardiac Surgery, The Pope John Paul II Province Hospital of Zamość, 22-400 Zamość, Poland; luke27@poczta.onet.pl; 4 Faculty of Health Sciences, Academy of Zamość, 22-400 Zamość, Poland

**Keywords:** ICD lead failure, ICD lead survival, reasons for ICD lead extraction, ICD lead malfunction

## Abstract

*Background and Objectives*: There is limited knowledge on ICD lead lifespan, the reasons for lead failure, and the influence of lead models. Our aim in this study was to compare the lifetime of individual lead models and the reasons for their extraction. *Materials and Methods*: We analyzed 3929 transvenous lead extractions (TLEs) (including 1068 ICD lead extractions). *Results*: The median age of an ICD lead removed (for all causes) was 61 months, three years shorter than that of PM leads. Old models with thick leads survived almost twice as long as thin and modern leads. ICD leads were removed due to infection (35.6%), mechanical damage (33.6%), and dysfunction (including perforation) (20.8%). Riata leads presented higher resistance to mechanical damage compared to Sprint Fidelis and Linox (dwell time medians; 124.0, 47.0 and 70.0 months). However, Durata leads presented higher resistance to mechanical damage compared to Sprint Fidelis and Linox (dwell time medians; 81.0, 68.0 and 70.0 months). Riata leads lasted almost twice as long as Sprint Fidelis. Linox leads lasted as long as theoretically fail-safe Sprint Quatro leads. *Conclusions*: 1. The median of ICD lead survival was three years shorter than that of PM leads. 2. ICD leads were most commonly removed due to infection, mechanical lead damage, and undamaged lead dysfunction (including perforation). 3. Old models of thick leads (Sprint, Ventritex, and Kainox) survived almost twice as long as thin leads from the transition period (Riata and Sprint Fidelis) and modern leads. Despite advances in the design of ICD leads, their lifetime has not changed significantly.

## 1. Introduction

There remains limited knowledge on ICD lead lifespan, reasons for ICD lead failure, and differences in the functionality of different lead models, as lead registers only record the occurrence of lead failure. In addition, a large percentage of ICD leads are replaced for other reasons. Transvenous lead extraction analyses focus mainly on the difficulties and dangers of extracting ICD leads and omit detailed analyses underlying the causes of removal or a lifetime comparison of individual lead models. To the best of our knowledge, this is the first detailed comparative study for virtually all models of implanted ICD leads, with a detailed analysis of the timing (from implantation to extraction) and reasons for extraction. Our analyses showed that many completely different leads from different manufacturers perform similarly; the observed differences were small or insignificant. Despite advances in the design of ICD leads during the last 20 years, the lifetime of such leads has not changed significantly, which suggests the role of factors other than the design of the lead. Our observations may indicate the important role of factors other than the quality of lead implantation. Therefore, it is important to pay attention to this problem.

## 2. Background

Patients are increasingly receiving ICD leads. Additionally, due to infections and dysfunctions, patients increasingly require ICD lead removal or replacement [1,2,3,4]. Transvenous lead extraction (TLE) plays a key role in lead management [5,6], with considerable literature on ICD lead survival [7,8,9,10,11,12,13,14,15,16,17,18,19,20]. These studies are based mainly on registers of implanted leads [12,13], multicenter analyses [8,11,15,16], and single center experiences [7,9,10,14,17,18,19,20]. Lead survival is presented as the percentage of leads still functioning well after 3, 5, and 6 years [10,15,18,20]; the appearance of lead failure during the follow-up period [16,17], or the annual appearance of lead failure [8,9], usually without a detailed analysis of the causes of dysfunction (infection and lead failure) or the method for dealing with the failed lead (lead replacement vs. abandonment).

Five-year survival of ICD leads has been estimated as 74–85% [10], 97.5 [15], 87.5% [18], and 95.3–97.4% [20], and lead failure incidence during 5 year FU has been estimated to be between 3.5% [17] and 11.0% [16]. The annual rate of lead failure incidence has been reported as 0.27% [8], 2.6% [9], and 0.54% [15]. A detailed analysis of the mechanisms and causes for lead failure was published in several reports [7,12,15,16,18,19,21,22,23].

Studies have focused on leads with more frequently reported failures (Rita, Sprint Fidelis, and Linox), usually comparing survival between models, as follows: Riata vs. Sprint Fidelis [9,14] and Linox vs. Sprint Quatro [14,17,18]. Survival differences between specific models from the same company have also been compared (e.g., Riata vs. Durata [8,16,18,19]). Despite the large quantity of literature on the difficulties and dangers of ICD lead extraction, these studies do not analyze the time of lead survival, explore the causes of lead failure (failure or non-damaged lead dysfunction), or offer a detailed analysis of indications for ICD lead extraction [1,2,3,4]. Only four reports compare the extraction efficiency and safety of leads considered potentially more dangerous or difficult to extract [21,22,23,24]. These studies focus on Sprint Fidelis and Riata leads and do not analyze lead duration or lead failure causes and mechanisms [7,12,21,22,23,24].

Using a 17-year-old TLE database with data on the extraction of over 1000 ICD leads (all models), we compared the lifetime of ICD leads (from implantation to extraction) alongside the reasons for extraction, taking into account the manufacturer and specific lead model.

## 3. Goal of the Study

The aim of the study was to comparatively analyze the functioning time of individual lead models (from implantation to extraction) and the reasons for their extraction. We compared the lifetime (from implantation to extraction) of different ICD lead models from different manufacturers and the detailed reasons for their extraction. The only additional purpose of the study was to compare the survival longevity of ICD and pacemaker leads (functioning in pacemaker systems).

## 4. Methods

### 4.1. Study Population

The study population consisted of 3929 patients, among whom 1068 had their ICD leads removed, whereas 2861 patients underwent pacing lead extraction (control group).

All 3929 reviewed transvenous lead extraction (TLE) procedures were performed between March 2006 and July 2023 by one very experienced operator at three high-volume centers.

### 4.2. Definitions

Indications for TLE, procedure effectiveness and complications were defined according to the recent TLE recommendations (2017 HRS consensus and 2018 EHRA guidelines) [5,6].

### 4.3. Reasons for ICD Lead Extraction

- Mechanical lead damage (electric failure)—A sudden increase in impedance >1500 Ohm or high-voltage impedance > 100 Ohm; >300 nonphysiological short interventricular-intervals (“crackles”) or SVVI with inappropriate discharge [10].
- Non-damaged lead dysfunction—Lead failure without mechanical damage: an exit/entry block (a linear increase in impedance > 1500 Ohm, high-voltage impedance > 100 Ohm, a linear decrease in sensing, or increase in the pacing threshold to an inacceptable level level), tip dislodgement, or extracardiac pacing.
- Lead heart wall perforation (dry or wet)—acute/subacute-symptomatic perforation (epicardial fluid or lead dysfunction); late—usually dry perforation—lead dysfunction or TLE due to other reasons and echocardiographic symptoms of perforation despite acceptable pacing sensing parameters.
- Prophylactic ICD lead replacement—TLE for other reasons (another lead extracted) and an ICD lead over 7–8 years of age in a (usually young) patient with a long life prognosis or recalled leads. TLE due to another reason (another lead extracted) and recalled lead.
- Other indication—An abandoned ICD lead, threatening/potentially threatening lead (loops or LDTVD), MRI indication, cancer, pocket pain, cessation of indication for ICD/CRT, recapture of venous access (symptomatic occlusion, SVC syndrome, or ICD lead replacement/upgrading), or technical reasons (conflict in type of connector, strong connection ICD lead with another one with scar causing impossible separation, or accidental functioning ICD lead damage in the generator region during another CIED procedure).
- Local pocket infection, systemic infection (with or without pocket infection)—Such removals were considered according to the EHRA international consensus document on how to prevent, diagnose, and treat cardiac implantable electronic device infections [25].

### 4.4. Dataset and Statistical Methods

#### Creation of the Subgroups for the Analysis of Events and Patients

**Figure 1:**
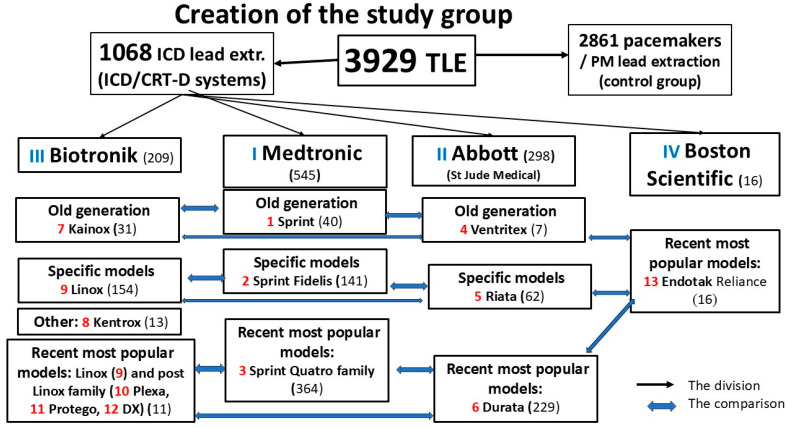
Creation of the study groups and subgroups.

All 3929 TLE procedures were divided into several groups and subgroups (Figure 1). The 1068 patients with removed ICD leads were subdivided according to the purpose of analysis, as depicted in Figure 1. For the purpose of this study, the TLE procedures/patients were divided by lead manufacturer (Medtronic, Abbott, Biotronik, and Boston Scientific) and then by lead design and production period (old generation, attempts at miniaturization, and modern period). The formation of the groups and subgroups of patients is presented in Figure 1. Some information (number of coils (single vs. dual), tip fixation mechanism (active vs. passive), and type of connector (Def1 vs. Def4) was omitted from this study because overly small subgroups would make a reliable statistical assessment impossible.

### 4.5. Statistics

Due to nonlinear distribution, continuous data are presented as the median and interquartile range. Continuous data in the first stage in all compared groups were analyzed using the ANOVA test, the results of which are presented in the relevant tables. In cases of statistical significance (<0.05), individual groups were compared with the Mann–Whitney U test, with values < 0.05 presented in the tables.

Categorical data are presented as both numbers and percentages. Similar to continuous data, in the first stage of analysis, all compared groups were analyzed using the Pearson’s Chi^2^ test, the results of which are presented in the tables. In cases of statistical significance (<0.05), individual groups were compared with the Chi^2^ test using Yates correction if appropriate, with values < 0.05 presented in the tables.

The Kaplan–Meier curves of time free from ICD lead failure (electric–mechanical lead damage or non-damaged lead failure) depending on the technology of lead construction were plotted and compared with a log rank test. To determine the risk factors for the electrical failure of ICD leads, Cox regression analysis was used. A two-tailed *p* value < 0.05 was considered statistically significant. Statistical analysis was performed using STATISTICA 13.1 PL (TIBCO, Poland, Cracow).

### 4.6. Approval of the Bioethics Committee

All patients gave their informed written consent to undergo TLE and use anonymous data from their medical records, as approved by the Bioethics Committee at the Regional Chamber of Physicians in Lublin no. 288/2018/KB/VII. The study was carried out in accordance with the ethical standards of the 1964 Declaration of Helsinki.

## 5. Results

In the study group of 1068 patients with an ICD lead, the median age was 62.13 years, with 18.91% women. Additionally, 35.49% of procedures were performed for infectious reasons. In the control group of 2861 individuals with a pacemaker, the median patient age was 67.30 years. In this group, 45.37% were women, and 29.61% of procedures were performed for infectious reasons. Table 1 present patient clinical characteristics, CIED systems and history of pacing, data on targeted leads of groups with ICD lead extraction, and pacemaker (PM) lead extraction for all patients/procedures.

Table 2 confirms the commonly known differences between patients with pacemakers (PMs) and those with ICD leads (ICD or CRT-D carriers). However, some information deserves further attention. Here, the median age of ICD lead removal (for all causes) is 61 months (5 years). Removed leads in PM patients were found to be three years older. Moreover, PM patients commonly undergo more CIED related procedures and more frequently have abandoned leads.

Extracted ICD leads were younger than PM leads (61 vs. 97 months) and more frequently removed due to infection (35.58 vs. 29.57%) or mechanical damage (33.61 vs. 24.50%), rather than dysfunction (8.52 vs. 13.53%) or another indication (6.84 vs. 23.21%).

The removed Biotronik leads were 18 months older than the leads from other companies. Such leads were removed less often for infectious reasons but almost twice as often due to mechanical damage (electric failure) (50.71 vs. 32.66 and 24.50%). Abbott leads dominated among the perforating leads (16.11 vs. 11.73; 7.66%).

Table 3 presents a comparison of the two groups of leads. The first consists of models of thin leads (Sprint Fidelis, Riata and Linox), while the second consists of older ICD lead models that were popular 15–20 years ago (Sprint, Ventritex, and Kainox–Kentrox).

Results of comparisons between lead models in each particular group are also presented.

It should be noted that the oldest models of ICD leads rarely function in ICD-V and CRT-D systems. Additionally, the distribution of TLE indications do not differ significantly between groups (Pearson’s Chi^2^ *p* = 0.066).

Riata leads were most often removed due to infection (40.32%), but Sprint Fidelis and Linox leads were most commonly removed due to lead damage (electric failure) (58.87 and 48.05%). Perforations were more common in Riata (14.52%) than Sprint Fidelis and Linox cases (4.26 and 8.44%). Sprint Fidelis was removed more often (12.06%) than Riata or Linox leads based on preventive indications (including lead recall) (4.84 and 1.95%).

Groups of patients with old models of ICD leads (Sprint, Ventritex, and Kainox–Kentrox) were smaller, making it more difficult to obtain statistical significance with noticeable differences in percentages. Notably, a higher percentage of mechanical lead damage was observed in patients with Ventritex leads (71.43%) than in those with Sprint and Kainox leads (37.50 vs. 56.82%). Among the old models of leads, perforations occurred mainly among Sprint leads (20.00%).

Table 4 presents an inter-group comparison of the most popular current models of ICD leads from three manufacturers: Medtronic (Sprint Quatro family), Abbott (Durata), and Biotronik (the Linox and post-Linox family: Protego Plexa and Linox Smart DX). The compared lead models functioned in similar types of CIED systems. Sprint Quattro was most often removed due to infection (48.35%), while those of the Linox family were most commonly removed due to mechanical lead damage (49.09%). However, Durata was most often removed due to lead dysfunction (including perforation) (28.82%).

The next part of the analysis concerns the survival time of different types of leads from the first implantation to extraction, both ICD leads of various manufacturers and pacemaker (PM) leads (Table 5). PM leads functioned significantly longer than ICD leads, regardless of the reason for extraction. For infection, the medians were 98 and 49 months, respectively; for mechanical lead damage, 120 and 70 months; for lead dysfunction (including perforation), 74 and 52 months; for other indications, 102 and 77 months; and and for any indications, 97 and 61 months. However, we found no statistically significant differences in the survival of ICD leads from individual manufacturers.

Table 6 presents a comparison of the survival longevity among two groups of leads. The first group consists of models using thin leads (Sprint Fidelis, Riata, and Linox), and the second consists of old ICD lead models that were popular 15-20 years ago (Sprint, Ventritex, and Kainox–Kentrox). The survival longevity between lead models in each particular group is also presented.

**Figure 2:**
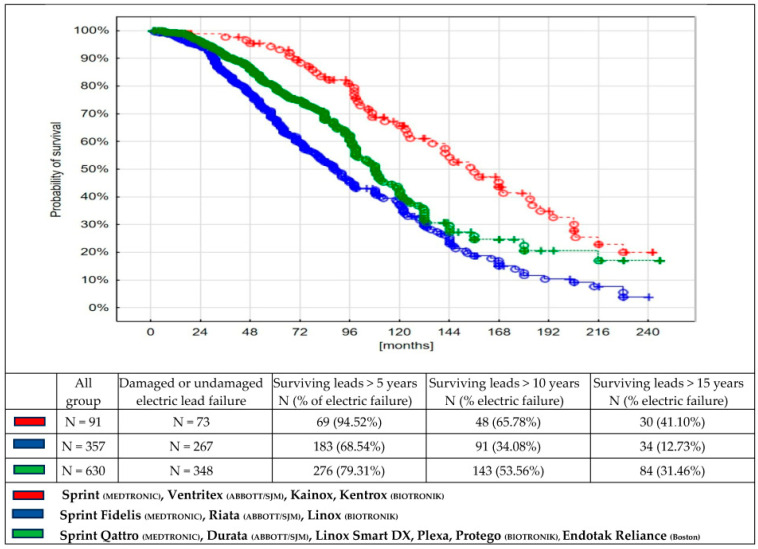
Time from implantation to transvenous lead extraction due to lead failure (electric–mechanical lead damage or non-damaged lead failure), depending on technology of lead construction: 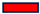 old—thick; 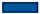 leads of the transition period; 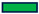 thin and modern leads, *p* < 0.001.

Notably, we observed a much longer survival period among thicker, older-type leads, given that thin leads are considered to be more prone to failure and more difficult to extract (Figure 2).

Riata leads were the last to be removed due mechanical damage when compared with Sprint Fidelis and Linox (medians 124.0, 47.00, and 70.00 months, respectively) or due to dysfunction of the undamaged lead (medians 74.00, 55.00, and 61.5 months, respectively). However, the differences in medians were not statistically significant. Additionally, lead survival time from implantation to extraction for any reason was the longest for Riata leads (medians 73,00, 55.00, and 65.00 months, respectively).

Among the old models of leads, Kainox–Kentrox leads had longer lifespans than Sprint and Ventritex leads. The median time for FIT-TLE due to lead dysfunction or perforation was 154.0 and 99.00 months (0.018) for Kainox–Kentrox and Sprint, respectively. The median time from implantation to extraction for any reason was 131.5, 99.00, and 125.0 months, respectively. However, these median differences were also not significant (Table 6).

Univariable Cox regression analysis showed that a younger patient age at the time of first implantation and the presence of ICD leads from the “transition period” (Sprint Fidelis _(MEDTRONIC)_, Riata _(ABBOTT/SJM)_, and Linox _(BIOTRONIK)_) were risk factors for ICD lead electric failure. This phenomenon was less frequent in the group of patients implanted with older types of electrodes (Sprint _(MEDTRONIC)_, Ventritex _(ABBOTT/SJM)_, Kainox, and Kentrox _(BIOTRONIK)_). The protective factors were older age and higher NYHA functional class. Multivariable Cox regression analysis showed that old ICD lead models (Sprint, Ventritex, and Kainox–Kentrox) had a 25.1% lower probability of electric failure occurrence year over year compared with the other leads (Table 7).

Table 8 compares the survival longevity between three of the most popular models of leads currently used by three manufacturers: Medtronic (Sprint Quatro family), Abbott (Durata), and Biotronik (the Linox and post-Linox family: Protego, Plexa and Linox Smart DX).

Systemic infections occurred latest in Sprint Quatro leads compared to Durata and Linox leads (medians of lead dwell times: 48.00, 42.00, and 45.00 months, respectively). However, Durata leads were more resistant to the mechanical failure than Sprint Fidelis and Linox leads (medians of dwell lead times: 81.00, 68.00, and 70.00 months, respectively). Linox leads were more resistant to non mechanical lead dysfunction than all other leads (medians of lead dwell times: 49.00, 47.00, and 47.00 months, respectively). However, the differences in the medians were not statistically significant. Moreover, lead survival time from implantation to extraction for any reason was only slightly longer for Linox leads (medians: 85.00, 81.00, and 63.00 months, respectively). However, all these median differences were not significant.

**Figure 3:**
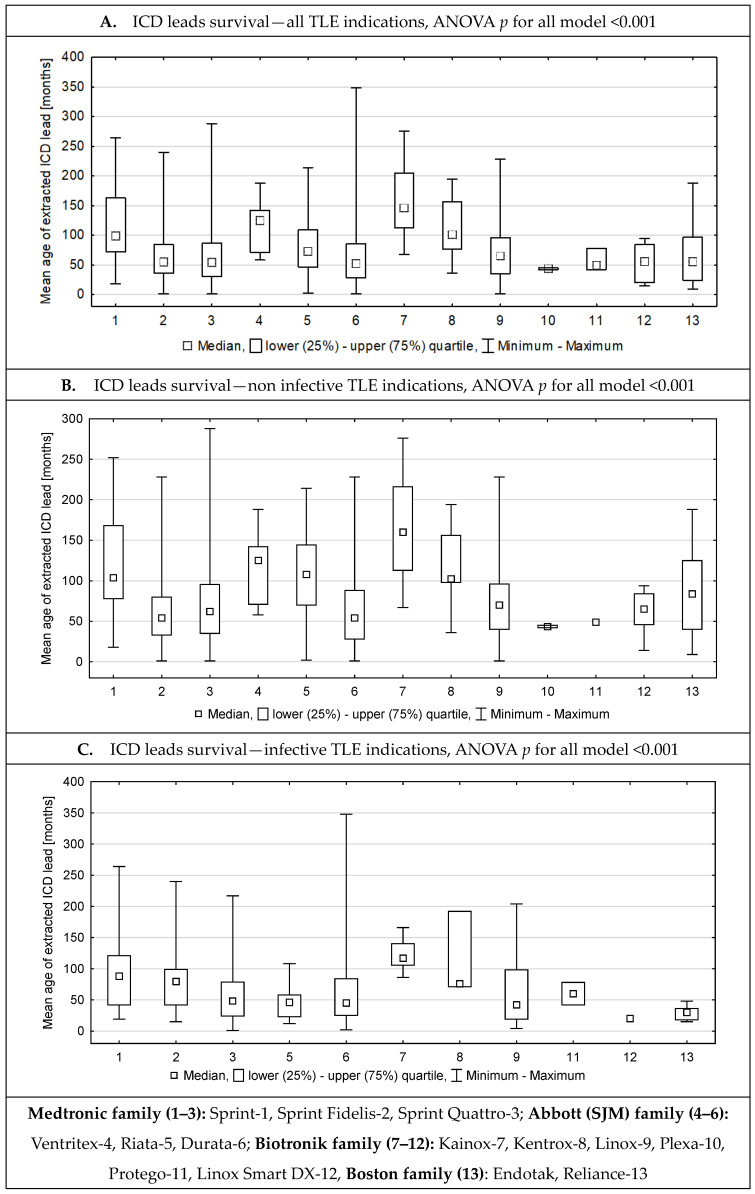
ICD lead survival—all TLE indications (**A**), all non-infective indications (**B**), and infective indications (**C**).

Figure 3 graphically presents the average survival time of individual lead models depending on the group of TLE causes (all, non-infectious, and infectious), with the life of the lead (scale) expressed in months. When analyzing the diagrams, the first three bars (1–3) are the Medtronic family, including Sprint-1, Sprint Fidelis-2, and Sprint Quattro-3; the next three bars (4–6) are the Abbott (SJM) family, including Ventritex-4, Riata-5, and Durata-6; the next group of bars (7–12) presents the Biotronik lead family, including Kainox-7, Kentrox-8, Linox-9, Plexa-10, Protego-11, and Linox Smart Dx DX-12; and the last bar (13) presents the Boston Scientific lead: Endotak, Reliance-13.

The first diagram (A) shows that the oldest (thick) leads functioned the longest, regardless of the manufacturer, although the Biotronik leads functioned the longest. Leads considered to be susceptible to damage had an intermediate time of operation, while modern leads offered the shortest time of operation, regardless of the manufacturer. However, this result is likely due to the methodology. Leads introduced to the market or implanted three or five years ago had no chance of showing longer survival in some patients.

The middle (B) diagram (removal for non-infectious reasons) provides additional information about the durability of the leads and their susceptibility to mechanical damage or dysfunction for other reasons. The general conclusions are similar (the oldest leads lasted the longest), but new observations also emerge. Potentially failing Riata leads lasted almost twice as long as potentially failing Sprint Fidelis leads. Linox leads, which are considered to have a higher risk of damage, lasted as long as the theoretically fail-safe Sprint Quatro leads, which are considered significantly less likely to fail.

The bottom (C) panel (diagram) shows how long the leads lasted until they were removed for infectious reasons. When interpreting the results, it should be remembered that these data were obtained from the TLE procedure database. In cases of early infections (up to a year), the leads were removed without the use of additional tools, and such patients and their procedures were not included in the TLE database. Thus, the figure presents the lifetime of ICD leads to the onset of delayed or late infection, which was much shorter (approximately 50 months) than the time to removal for non-infectious reasons.

## 6. Discussion

There is considerable literature on ICD lead survival [7,8,9,10,11,12,13,14,15,16,17,18,19,20,21,22,23,24,25]. The five-year survival of ICD leads was previously estimated to be 74–85% [10], 97.5 [15], 87.5% [18], and 95.3–97.4% [20]. Additionally, lead failure incidence during five-year FU was estimated to be 3.5% [17] and 11.0% [16]. The annual rate of lead failure incidence was reported as 0.27% [8], 2.6% [9], and 0.54% [15]. In this study, we showed that the median age of an ICD lead removed (for all causes) is 61 months (5 years), three years shorter than that of PM leads. This result is consistent with the observations of other authors that lead failures occur more frequently starting from the fifth year of operation of the lead, with the frequency of failure increasing significantly after 7 and 8 years [10,14,16,19,20]. Few studies on lead survival have focused on assessing one type of lead, e.g., Riata [22], Durata [15], or Linox [12]. Most studies have, instead, compared two types of leads, e.g., Riata vs. Durata [8,16,19], Sprint Fidelis vs. Riata [11,23], Sprint Quatro vs. Linox [17], or three models simultaneously [14,18]. The cited studies did not show any significant differences between the products of different manufacturers or the durability of models from the same company, although some differences were noted.

We showed that Riata was most often removed due to infection (40.32%), whereas Sprint Fidelis and Linox were removed due to lead damage (electric failure) (58.87 and 48.05%). This observation is consistent with the observations of Richasson et al. [23].

Perforations in our observations were more common in Riata cases (14.52%) than in Sprint Fidelis and Linox cases (4.26 and 8.44%). In other reports, the issue of perforation was omitted or treated marginally. In our studies, a thorough echocardiographic examination (TTE and TEE) was mandatory for the lead extraction procedure. We also reported asymptomatic cases of tip penetration into the pericardial fat and showed that ICD leads, compared with PM leads, were removed more frequently for infection (35.58%) and mechanical damage (33.61%) and less frequently due to lead dysfunction (8.52%) or other indications (6.84%). The present results can be compared with those of the GALAXY Registry [12]. This study tracked 3933 leads and found that the most common adverse events were oversensing (0.6%), conductor fracture (0.3%), failure to capture (0.3%), lead dislodgement (0.3%), insulation breach (0.3%), and abnormal pacing impedance (0.2%); the cumulative survival probability was 96.3% at 5 years [12]. Other authors focused on selected lead failure mechanisms. Valk et al. examined the long-term performance of 374 leads from the Riata family. Electrical abnormalities (mainly noise; 65%) were observed in 7.8% of patients [22]. Kleemann compared the performance of 1407 Durata and Riata leads and reported no differences in the defect rates after 5 and 10 years (13% and 38%). Major causes of lead failure were compression of the lead in the clavicular region, generator to lead friction, and distal fatigue fracture [16]. Cairns et al. analyzed three prospective registries, enrolling 11,155 patients. Over 4.6 years, the authors observed 1.53% mechanical failures and 0.62% electrical dysfunctions determined to represent nonmechanical failure [19]. O’Connor et al. examined the survival rates of Sprint Quattro, Endotak Reliance, and Linox (287) ICD leads, noting that the predominant abnormality was the detection of nonphysiological electrical signals [14]. Marai examined the performance of Linox and Sprint Quatro 340 leads. In total, 3.5% met the criteria for lead failure within 61.2 months; all abnormalities were considered together. Noise with inappropriate ventricular arrhythmias, with or without therapy, were most common (83%). In addition, high pacing thresholds and high impedances were detected rarely (17%) [17].

Our analysis of survival longevity showed that Riata leads were the last to be removed due to mechanical damage (medians 124.0 and 70.00 months, respectively) or dysfunction of the undamaged lead (medians 74.00 and 61.50 months, respectively) when compared with Sprint Fidelis and Linox. Additionally, the lead survival time from implantation to extraction for any reason was only slightly longer for Linox leads (median 85.00, 81.00, and 63.00 months, respectively). However, all these median differences were not significant.

Potentially failing Riata leads lasted almost twice as long as potentially failing Sprint Fidelis leads. Linox leads, which are considered to have a higher risk of damage, lasted as long as the theoretically fail-safe Sprint Quatro leads, which are considered significantly less likely to fail.

Although the present study could not scientifically confirm this finding, our results indicate a limited lifespan of at least some ICD leads. This outcome suggests the value in considering ICD lead replacement when replacing the generator. The extraction of younger leads is relatively safe, with patient discharge possible during the same day, or the day following, a TLE procedure [26].

## 7. Conclusions

1. The median ICD lead survival is three years shorter than that of PM leads.
2. ICD leads are most commonly removed due to infection, mechanical lead damage, or undamaged lead dysfunction (including perforation).
3. Old models of thick leads (Sprint, Ventritex, and Kainox) lasted almost twice as long as thin leads from the transition period (Riata and Sprint Fidelis) and modern leads.
4. Despite advances in the design of ICD leads, the lifetime of leads has not changed significantly, which indicates the role of factors other than the design of the lead.

### Study Limitations

One key limitation due to the focus of the present study lies in our use of information on ICD leads from a large database of transvenous lead extraction procedures. This database contains detailed information on all reasons for removing leads, not just removal due to infection or lead failure. There are many reasons why a functional, uninfected lead might be removed. The advantage of using such materials for research on ICD leads is that, in addition to information on current measurements of the stimulation parameters and memory of the unit, these data offer fairly accurate radiological images and the ability to visualize (i.e., macroscopic examination) the removed lead on the operating table. The limitation of our research is the lack of a subsequent thorough examination of the removed leads to detect non-visible damage to the conductors or the insulation separating the individual conductors.

The main difference between our work and the current literature is that this study analyzed lead removals, allowing us to accurately assess how long after implantation and for what reasons the leads were removed. However, we do not have information about all leads implanted in the region from which we receive patients or the subsequent fates of the leads and patients.

An additional advantage of this study is that it includes all models of ICD leads implanted in the last 25 years, including the oldest models. These older models have been unavailable on the market for years but remained in patients until recently (Sprint, Ventritex, and Kainox leads).

We did not systematically analyze the mechanisms and causes of mechanical damage to the leads, which would have been outside the scope of the present study. It was not the purpose of this study to analyze the influence of mechanical issues as potential lead failure risk factors, such as the number of coils, the mechanism of electrode tip fixation, and the type of connector or electrode insertion route.

## Figures and Tables

**Table 1:** Demographic, clinical, and system data in all examined patient groups.

	ICD Lead Extraction Among ICD/CRT-D Group	Pacemaker Lead Extraction Among PM Group (Systems)	All Patients/Procedures
	N (%), Median [IQR]	N (%), Median [IQR]	N (%), Median [IQR]
Number of patients	1068 (100.0)	2861 (100.0)	3929 (100.0)
Female	202 (18.91)	1298 (45.37) <0.001	1500 (38.18)
Prior myocardial infarction	481 (35.11)	375 (13.11) <0.001	847 (21.57)
Left ventricle ejection fraction [%]	35.00 [24.00]	57.00 [15.00] <0.001	54.00 [23.00]
NYHA functional class III or IV	380 (35.58)	383 (13.39) <0.001	763 (19.42)
Charlson index (points)	4 [6]	4 [4] 0.003	4 [4]
Pacemaker	0 (0.00)	2861 (100.0)	2861 (72.82)
ICD	831 (77.81)	0 (0.00)	824 (20.97)
CRT-D	237 (22.19)	0 (0.00)	237 (6.03)
Local (pocket infection)	105 (9.83)	266 (9.30) 0.496	371 (9.45)
Systemic infection	275 (25.75)	580 (20.27) <0.001	855 (21.76)
Age of oldest lead [years]	5.08 [5.17]	8.25 [8.50] <0.001	7.17 [7.50]
Age of all leads in the patient [years]	4.83 [4.67]	7.92 [7.75] <0.001	7.00 [7.25]
Age of ICD leads [months]	61.00 [63.00]		
Age of PM leads [months]		97.00 [102.0] <0.001	
Number of prior CIED procedures	1 [1]	2 [1] <0.001	2 [1]
Two or more prior CIED procedures	522 (48.88)	1553 (54.28) <0.001	2075 (52.81)
Presence of an abandoned lead	85 (7.96)	328 (11.46) <0.001	413 (10.51)

ICD—implantable cardioverter defibrillator; CRT-D—cardiac resynchronization therapy defibrillator; PM—pacemaker; IQR—interquartile range; NYHA—New York Heart Association; CIED—cardiac implantable electronic device.

**Table 2:** Reasons for lead extraction in patients with ICD leads from different manufacturers.

	Medtronic ICD Leads	Abbott (SJM) Leads	Biotronik Leads	Boston Leads	ANOVA, Pearson’s Chi^2^	All ICD Leads	All Leads Among PM Systems Only “U” Mann–Whitney. Chi^2^
Subgroup number	I	II	III	IV			
Number of patients in compared groups	545	298	209	16		1068	2861
	Median [IQR] N (%)	Median [IQR] N (%)	Median [IQR] N (%)	Median [IQR] N (%)		Median [IQR] N (%)	Median [IQR] N (%)
Extracted lead age [months]	58.00 [56.00]	58.00 [63.00]	74.00 [76.00] <0.001^(vs I)^ <0.001^(vs II)^	55.00 [72.00]	<0.001	61.00 [63.00]	97.00 [102.0] <0.001
ICD lead as a component of the CRT-D system	118 (21.65)	76 (25.50)	39 (18.66)	4 (25.00)	0.312	237 (22.19)	NA
TLE due to system infection (local or systemic)	205 (37.61)	113 (37.80)	57 (27.27) 0.008^(vs I)^ 0.012^(vs II)^	5 (31.25)	0.044	380 (35.58)	846 (29.57) <0.001
TLE due to mechanical lead damage (electric failure)	178 (32.66)	73 (24.50)	106 (50.71) <0.001^(vs I)^ <0.001^(vs II)^	2 (12.50)	<0.001	359 (33.61)	701 (24.50) <0.001
TLE due to lead dysfunction (lead failure without mechanical damage)	43 (7.90)	30 (10.07)	16 (7.66)	2 (12.50)	0.633	91 (8.52)	387 (13.53) <0.001
TLE due to lead heart perforation (dry or wet)	64 (11.73)	48 (16.11)	16 (7.66) 0.003^(vs II)^	3 (18.75)	0.030	131 (12.27)	264 (9.22) 0.006
TLE due to lead dysfunction (including perforation)	107 (19.63)	78 (26.18) 0.028^(vs I)^	32 (15.32) 0.003^(vs II)^	5 (31.25)	0.014	222 (20.79)	651 (22.75)
Prophylactic functional ICD lead replacement (via another TLE)	21 (3.85)	8 (2.68)	4 (1.91)	1 (6.25)	0.455	34 (3.18)	0 (0.00) <0.001
TLE due to other indication (abandoned lead, threatening lead, MRI, cancer, pain, superfluous system, recapture of venous access, or technical reasons)	34 (6.24)	26 (8.72)	10 (4.78)	3 (18.75)	0.074	73 (6.84)	664 (23.21) <0.001

SJM—St Jude Medical; TLE—transvenous lead extraction; IQR—interquartile range; ICD—implantable cardioverter defibrillator; PM—pacemaker. Medtronic leads: Sprint, Sprint Fidelis, Sprint Quattro family (6935, 6946, 6947); Abbott (SJM) leads; Ventritex, Riata, Riata ST^®^, Durata; Biotronik leads: Kainox, Kentrox, Linox, DX, Plexa, Protego; Boston Scientific leads: Endotak Reliance.

**Table 3:** Reasons for extracting specific models of ICD lead and old models of ICD leads.

	Group 1				Group 2				Group 1 (G1)	Group 2 (G2)	
	Sprint Fidelis	Riata^®^, Riata ST^®^	Linox	Pear-son’s Chi^2^	Sprint	Ventritex	Kainox, Kentrox	Pearson’s Chi^2^			Chi^2^ test
Number of subgroups	2	5	9	2, 5, 9	1	4	7–8	1, 4, (7–8)	2, 5, 9	1, 7, 7–8	G1 vs. G2
	N (%)	N (%)	N (%)	P	N (%)	N (%)	N (%)	P	N (%)	N (%)	*p*
Number of patients	141 (100.0)	62 (100.0)	154 (100.0)		40 (100.0)	7 (100.0)	44 (100.0)		357 (100.0)	91 (100.0)	
ICD-V	75 (53.19)	35 (56.45)	72 (46.75)	0.346	19 (47.50)	4 (57.14)	22 (50.00)	0.890	182 (50.98)	45 (49.45)	0.886
ICD-D	48 (34.04)	19 (30.65)	48 (31.17)	0.834	17 (42.50)	3 (42.86)	20 (45.45)	0.962	115 (32.21)	40 (43.96)	0.048
CRT-D	18 (12.77)	8 (12.90)	34 (22.08)	0.068	4 (10.00)	0(0.00)	2 (4.54)	0.586 (Y)	60 (16.81)	6 (6.59)	0.014
TLE due to system infection (local or systemic)	22 (15.60)	25 (40.32) <0.001^(vs 2)^	43 (27.92)	<0.001	7 (17.50)	0(0.00)	11 (25.00)	0.403 (Y)	90 (25.21)	18 (19.78)	0.280
TLE due to mechanical lead damage (electric failure)	83 (58.87)	22 (35.48) 0.002^(vs 2)^	74 (48.05)	0.007	15 (37.50)	5 (71.43)	25 (56.82)	0.101	179 (50.14)	45 (49.45)	0.907
TLE due to lead dysfunction (lead failure without mechanical damage)	9 (6.38)	2 (3.23)	15 (9.74)	0.217	4 (10.00)	0(0.00)	1 (2.27)	0.135 (Y)	26 (7.28)	5 (5.49)	0.607
TLE due to lead heart perforation (dry or wet)	6 (4.26)	9 (14.52) 0.002^(vs 2)^	13 (8.44)	0.041	8 (20.00)	0(0.00)	4 (9.09)	0.154 (Y)	28 (7.84)	12 (13.19)	0.111
TLE due to lead dysfunction (including perforation)	15 (10.64)	11 (17.75)	28 (18.18)	0.160	12 (30.00)	0(0.00)	5 (11.36)	0.064 (Y)	54 (15.13)	17 (18.68)	0.297
Prophylactic functional ICD lead replacement (via another TLE)	17 (12.06)	3 (4.84)	3 (1.95) <0.001^(vs 2)^	0.002	1 (2.50)	0(0.00)	1 (2.27)	0.946 (Y)	23 (6.44)	2 (2.20)	0.115
TLE due to other indications (abandoned lead, threatening lead, MRI, cancer, pain, superfluous system, recapture of venous access, or technical reasons)	4 (2.84)	1 (1.61)	6 (3.90)	0.664	5 (12.50)	1 (14.29)	3 (6.82)	0.630	11 (3.08)	9 (9.89)	0.005

TLE—transvenous lead extraction; ICD—implantable cardioverter defibrillator; MRI—magnetic resonance imagination; (Y)—Chi^2^ test with Yates correction.

**Table 4:** Reasons for extraction among the most popular recent lead models.

	Medtronic; Sprint Quattro Family (6935, 6946, 6947)	Abbott (SJM); Durata	Biotronik: Linox, Linox Smart DX, Plexa, Protego	Pearson Chi^2^	All Patients with ICD Leads
Number of subgroups	3	6	9–12	3 vs. 6 vs. (9–12)	
	N (%)	N (%)	N (%)		N (%)
Number of patients/ICD leads (%)	364 (100.0)	229 (100.0)	165 (100.0)		758 (100.0)
ICD-V	146 (40.11)	84 (36.68)	79 (47.88)	0.078	309 (40.77)
ICD-D	122 (33.52)	77 (36.84)	49 (29.70)	0.646	248 (32.72)
CRT-D	96 (26.37)	68 (32.54)	37 (22.42)	0.312	201 (26.52)
TLE due to infection	176 (48.35)	88 (38.42) 0.029^(vs 3)^	46 (27.88) <0.001^(vs 3)^ <0.001^(vs 6)^	<0.001	310 (40.90)
TLE due to mechanical lead damage (electric failure)	80 (21.98)	46 (20.09)	81 (49.09) <0.001^(vs 3)^ <0.001^(vs 6)^	<0.001	207 (27.31)
TLE due to lead dysfunction (including perforation) (lead failure without mechanical damage)	80 (21.98)	66 (28.82)	28 (16.97) <0.001^(vs 6)^	0.018	174 (22.96)
Prophylactic functional ICD lead replacement (via another TLE)	3 (0.82)	5 (2.18)	3 (1.82)	0.365	11 (1.45)
TLE due to other indications (abandoned lead, threatening lead, MRI, cancer, pain, superfluous system, recapture of venous access, or technical reasons)	25 (6.87)	24 (10.48)	7 (4.24)	0.057	56 (7.39)

SJM—St Jude Medical; TLE—transvenous lead extraction; ICD—implantable cardioverter defibrillator (V—single chamber; D—dual chamber); CRT-D—cardiac implantable resynchronization therapy defibrillator; MRI—magnetic resonance imagination.

**Table 5:** The longevity of lead survival (all manufacturers, all models) depending on the reason for ICD lead extraction (from implantation to ICD lead extraction).

	Medtronic; Sprint, Sprint Fidelis, Sprint Quattro Family (6935, 6946, 6947)	Abbott (SJM); Ventritex, Riata, Riata ST^®^, Durata	Biotronik; Kainox, Kentrox, Linox, DX, Plexa Protego	Boston; Endotak Reliance	ANOVA I ÷ IV	All Patients with ICD Leads	All Patients with Sensing/Pacing Leads Among PM Systems Only
	I	II	III	IV			Mann-Whitney “U” test ICD vs. PM
Number of patients (%)	545 (51.03)	298 (27.90)	209 (19.57)	16 (1.81)		1068 (100.0)	2861 (100.0)
	Median [IQR]	Median [IQR]	Median [IQR]	Median [IQR]		Median [IQR]	Median [IQR]
Time–FIT–TLE due to infection [months]	50.00 [59.00]	45.00 [54.00]	66.00 [80.00]	30.00 [48.00]	0.103	49.00 [59.5]	98.00 [92.00] <0.001
Time–FIT–TLE due to mechanical lead damage (electric failure) [months]	60.50 [64.00]	96.00 [67.00]	82.00 [70.00]	98.00 [116.0]	0.968	70.0 [67.00]	120.0 [131.5] <0.001
Time–FIT–TLE due to lead dysfunction (including perforation) (lead failure without mechanical damage) [months]	52.00 [50.00]	46.50 [55.00]	68.50 [73.5]	74.00 [90.00]	0.179	52.00 [56.00]	74.00 [82.00] <0.001
Time–FIT–TLE Prophylactic functional ICD lead replacement (via another TLE) [months]	80.00 [45.00]	116.0 [89.00] <0.001^(vs I)^	82.50 [48.00]	188.0 *	0.002	90.50 [57.00]	AC
Time–FIT–TLE due to other indications (abandoned lead, threatening lead, MRI, cancer, pain, superfluous system, recapture of venous access, or technical reasons) [months] [months]	83.50 [82.00]	65.00 [51.00]	131.5 [91.00]	84.00 [23.00]	0.217	77.00 [76.00]	102.0 [102.0] <0.001
Time–FIT–TLE due to any indications	58.00 [56.00]	58.00 [63.00]	74.00 [76.00]	55.00 [72.50]	0.115	61.00 [63.00]	97.00 [102.0] <0.001

TLE—transvenous lead extraction; IQR—interquartile range; ICD—implantable cardioverter defibrillator; PM—pacemaker; Time-FIT TLE—time from implantation to transvenous lead extraction; *—one case; AC—any cases; MRI—magnetic resonance imagination.

**Table 6:** ICD lead survival for specific models and the reasons for ICD lead extraction.

	Group 1				Group 2				Group 1 (G1)	Group 2 (G2)	
	Sprint Fidelis	Riata	Linox	ANO-VA	Sprint	Ventritex	Kainox, Kentrox	ANOVA			Mann-Whitney “U” test
Number of subgroups	2	5	9	2, 5, 9	1	4	7–8	1, 4, 7–8	2, 5, 9	1, 4, 7–8	G1 vs. G2
Number of patients	141	62	54		40	7	44		357	91	
	Median, [IQR]	Median, [IQR]	Median, [IQR]	P	Median, [IQR]	Median, [IQR]	Median, [IQR]	P	Median, [IQR]	Median, [IQR]	P
ICD-V (Time–FIT–TLE) [months]	52.00 [52.00]	77.00 [76.00]	69.00 [65.00]	0.845	104.0 [88.00]	124.0 [83.00]	148.0 [82.00]	0.263	62.00 [61.00]	124.0 [82.00]	<0.001
ICD-D (Time–FIT–TLE) [months]	57.00 [40.50]	73.00 [65.00]	62.50 [66.50]	0.212	94.00 [92.00]	125.0 [65.00]	112.5 [83.50]	0.232	61.00 [53.00]	108 [85.00]	<0.001
CRT-D (Time–FIT–TLE) [months]	83.50 [57.00]	56.00 [57.00]	58.00 [55.00]	0.467	114.5 [98.50]	AC	99.00 [46.00]	0.354	58.00 [58.00]	114 [39.00]	0.015
Time–FIT–TLE due to infection [months]	79.50 [57.00]	46.00 [35.00]	42.00 [79.00]	0.560	88.00 [79.00]	AC	112.0 [62.00]	0.160	50.00 [61.00]	105 [56.00]	<0.001
Time–FIT–TLE due to mechanical lead damage [months]	47.00 [37.00]	124.0 [64.00]	70.00 [53.00]	0.846	104.0 [82.00]	136.0 [17.00]	142.0 [96.00]	0.314	61.00 [61.00]	124.0 [88.00]	<0.001
Time–FIT–TLE due to lead dysfunction (including perforation) [months]	55.00 [57.00]	74.00 [43.00]	61.50 [49.50]	0.701	99.00 [47.00]	AC	154.0 [51.00]	0.018	63.00 [56.00]	108.0 [68.00]	<0.001
Time–FIT–TLE due to prophylactic functioning lead replacement [months]	72.00 [35.00]	80.00 [70.00]	69.00 [61.00]	0.109	179.0 *	AC	104.0 *	1.00	72.00 [35.00]	141.0 [75.00]	0.098
Time–FIT–TLE due to other indication [months]	73.50 [58.50]	62.00 *	108.5 [91.00]	0.893	169.0 [121.0]	71.00 *	160.0 [111.0]	0.764	84.00 [70.00]	160.0 [121.00]	0.119
Time–FIT–TLE due to any indications [months]	55.00 [48.00]	73.00 [63.00]	65.00 [61.00]	0.187	99.00 [91.50]	125.0 [71.00]	131.5 [78.00]	0.106	61.00 [60.00]	117.0 [86.00]	<0.001

TLE—transvenous lead extraction; IQR—interquartile range; ICD—implantable cardioverter defibrillator (V; single chamber; D; dual chamber); CRT-D—cardiac implantable resynchronization therapy defibrillator; Time–FIT–TLE—time from implantation to transvenous lead extraction; *—one case; AC—any case.

**Table 7:** Factors influencing ICD lead electric failure (mechanical lead damage or non-damaged lead failure) based on results of the uni-and multivariable Cox regression model.

	Univariable Cox Regression			Multivariable Cox Regression		
	HR	95% CI	*p*	HR	95% CI	*p*
Female [y/n]	0.989	0.812-1.204	0.911			
Age of patient during first implantation [by 1 year]	1.013	1.007–1.019	<0.001	1.265	1.242–1.289	<0.001
Age of patient during TLE [by 1 year]	0.991	0.985–0.996	<0.001	0.812	0.800–1.029	<0.001
Left ventricle ejection fraction [by 1%p]	0.999	0.994–1.004	0.647			
NYHA FC [by 1]	0.815	0.726–0.916	<0.001	0.520	0.452–0.598	<0.001
Number of leads in the system [by one]	0.831	0.748–0.923	<0.001	0.918	0.820–1.028	1.138
Old and thick models of ICD leads [y/n]	0.494	0.378–0.645	<0.001	0.749	0.562–0.996	0.048
ICD leads from transition period [y/n]	1.288	1.091–1.521	0.003	1.132	0.955–1.343	0.152
Thin and modern ICD leads [y/n]	0.863	0.714–1.043	0.128			

ICD—implantable cardioverter defibrillator; TLE—transvenous lead extraction; %p—percentage points; NYHA FC class—New York Heart Association functional class; old and thick ICD lead models models—Sprint _(MEDTRONIC)_, Ventritex _(ABBOTT/SJM)_, Kainox, Kentrox _(BIOTRONIK)_; ICD leads of transition period—Sprint Fidelis _(MEDTRONIC)_, Riata _(ABBOTT/SJM)_, Linox _(BIOTRONIK)_; thin and modern ICD leads—Sprint Qattro _(MEDTRONIC)_, Durata _(ABBOTT/SJM)_, Linox Smart DX, Plexa, Protego _(BIOTRONIK),_ Endotak Reliance _(Boston)_.

**Table 8:** Longevity of survival between the most popular ICD lead models.

Popular Models of ICD Lead Survival and the Reason for ICD Lead Extraction	Medtronic: Sprint Quatro Family: (6935, 6946, 6947)	Abbott (SJM): Durata	Biotronik: LINOX + (DX, Plexa, Protego)	ANOVA	All Patients with ICD Leads
Number of subgroups	3	6	9–12		
Number of patients (%)	364 (48.02)	229 (30.21)	165 (21.77)		758 (100.0)
	Median [IQR]	Median [IQR]	Median [IQR]		Median [IQR]
Time–FIT–TLE due to infection [months]	48.00 [54.00]	45.00 [59.00]	42.00 [58.00]	0.999	47.00 [58.00]
Time–FIT–TLE due to mechanical lead damage (electrical failure) [months]	68.00 [68.00]	81.00 [50.00]	70.00 [51.00]	0.778	70.00 [55.00]
Time–FIT–TLE due to lead dysfunction (including perforation) [months]	49.00 [47.00]	40.00 [47.00]	61.50 [49.50]	0.025	46.00 [50.00]
Time–FIT–TLE due to prophylactic indications [months]	175.0 [85.50]	154.0 [46.00]	69.00 [61.00]	0.174	58.00 [58.00]
Time–FIT–TLE due to other indications [months]	81.00 [74.00]	63.00 [52.50]	85.00 [131.0]	0.057	73.50 [55.00]
Time–FIT–TLE due to any indications [months]	54.00 [56.00]	52.00 [57.00]	64.00 [59.00]	0.027	55.00 [57.00]

SJM—St Jude Medical; TLE—transvenous lead extraction; IQR—interquartile range; ICD—implantable cardioverter defibrillator; Time–FIT–TLE—time from first implantation to transvenous lead extraction.

## Data Availability

Readers can access the data supporting the conclusions of this study at www.usuwanieelektrod.pl.

## Abbreviations

CIED	cardiac implantable electronic device
CRT-D	cardiac resynchronization therapy implantable defibrillator
EHRA	European Heart Rhythm Association
HRS	Heart Rhythm Society
ICD	implantable cardioverter-defibrillator
TLE	transvenous lead extraction
